# Supplementary material for: Maternal Obesity and Kawasaki Disease-like Vasculitis: A New Perspective on Cardiovascular Injury and Inflammatory Response in Offspring Male Mice
Source: Nutrients. 2023 Aug 31;15(17):3823. doi: 10.3390/nu15173823 (PMC10490206; doi:10.3390/nu15173823)
Supplement: Supplementary file 1 [file nutrients-15-03823-s001.zip › nutrients-2576960-supplementary.pdf]

Supplementary Table S1. The sequences of primers for qRT-PCR (5'-3').

Genes	Sequences (5'-3')
Mouse *Eotaxin*	F: TCCATCCCAACTTCCTGCTGCT
	R: CTCTTTGCCCAACCTGGTCTTG
Mouse *Csf2*	F: AACCTCCTGGATGACATGCCTG
	R: AAATTGCCCCGTAGACCCTGCT
Mouse *IL-1α*	F: ACGGCTGAGTTTCAGTGAGACC
	R: CACTCTGGTAGGTGTAAGGTGC
Mouse *IL-1β*	F: TGGACCTTCCAGGATGAGGACA
	R: GTTCATCTCGGAGCCTGTAGTG
Mouse *IL-9*	F: TCCACCGTCAAAATGCAGCTGC
	R: CCGATGGAAAACAGGCAAGAGTC
Mouse *IL-12α*	F: ACGAGAGTTGCCTGGCTACTAG
	R: CCTCATAGATGCTACCAAGGCAC
Mouse *IL-12β*	F: TTGAACTGGCGTTGGAAGCACG
	R: CCACCTGTGAGTTCTTCAAAGGC
Mouse *Kc*	F: TCCAGAGCTTGAAGGTGTTGCC
	R: AACCAAGGGAGCTTCAGGGTCA
Mouse *Rantes*	F: CCTGCTGCTTTGCCTACCTCTC
	R: ACACACTTGGCGGTTCCTTCGA
Mouse *Tnf-α*	F: CTCTTCTGCCTGCTGCACTTTG
	R: ATGGGCTACAGGCTTGTCACTC
Mouse *Actb*	F: CATTGCTGACAGGATGCAGAAGG
	R: TGCTGGAAGGTGGACAGTGAGG
